# Complete mitochondrial genome of *Urocissa erythroryncha* (Passeriformes: Corvidae)

**DOI:** 10.1080/23802359.2018.1481782

**Published:** 2018-06-18

**Authors:** Ruihua Liu, Renrui Chen, Jie Liu, Yonghong Xiong, Xianzhao Kan

**Affiliations:** a The institute of Bioinformatics, College of Life Sciences, Anhui Normal University, Wuhu, China;; b The Xinyu Pharmaceutical Co. Ltd., Suzhou, China

**Keywords:** *Urocissa erythroryncha*, mitochondrial genome, phylogenetic analysis

## Abstract

The complete mitochondrial genome of *Urocissa erythroryncha* is 16930 bp in length. It was predicted to contain 13 PCGs, 22 *tRNA* genes, and 2 *rRNA* genes, and a putative control region. All of the PCGs initiated with ATG, except for *MT-COX1* which began with GTG and *MT-ND3* began with ATA, while stopped by three types of stop codons. Phylogenetic analysis showed that *Urocissa erythroryncha* and the other species of Corvidae were monophyletic group in this study. And the monophyly of the genus *Pyrrhocorax* was strongly supported. Moreover, our results also support a sister-group relationship between Corvidae and Muscicapidae.

Passeriformes is the largest order of Aves (Clements et al. 2016). The birds generally known as corvids is a widespread family named Corvidae (Aves: Passeriformes) (Gill and Donsker 2018). In our study, an adult *Urocissa erythroryncha* specimen was caught from Wuhu (geographic coordinate: 31.33°N, 118.38°E), Anhui Province, southeastern of China. The specimen was preserved in ethanol and stored to Anhui Normal University, under the voucher number Kan-K0024. Through the standard phenol/chloroform methods, the total genomic DNA of the *Urocissa erythroryncha* (code Kan-K0024) was extracted from the muscle tissue. The fourteen PCR and sequencing primers specific to *U. erythroryncha* mitochondrial genome were designed, and two long overlapping fragments were amplified to avoid the possibility of nuclear copies of mitochondrial genes (Kan et al. 2010; Zhang L et al. 2012; Zhang Q et al. 2017).

We obtained the complete mitochondrial genome of *U. erythroryncha* (GenBank Accession NC_020426) in this study. We described 16930 bp of *U. erythroryncha* mitochondrial genome DNA, including 13 protein-coding genes (PCGs), 22 transfer RNA (tRNA) genes, 2 ribosomal RNA (rRNA) genes, and 1 putative control region. All *tRNA* genes can fold into a typical cloverleaf second structure. All of the PCGs initiated with ATG, except for *MT-COX1* which began with GTG. And meanwhile three types of termination codons were identified. AGG for *MT-COX1*; TAA for *MT-ND2*, *MT-COX2*, *MT-ATP6*, *MT-ATP8*, *MT-ND3*, *MT-ND4L*, *MT-ND6*, and *MT-CYTB*; AGA for *MT-ND1* and *MT-ND5*; and incomplete stop codon T- for *MT-ND2*, *MT-COX3*, and *MT-ND4*. We can detect one single control region (D-loop) between *tRNA^Glu^* and *tRNA^Phe^*. The D-loop region is 1346 bp in length. The overall base composition of the mitochondrial genome is as follows: A (30.92%), T (24.66%), G (14.32%), C (30.10%), with the A + T content of 55.58%. Nucleotide composition was calculated by MEGA v6.06 (Tamura et al. 2013). AT and GC skews were calculated by using the formulas (A – T)/(A + T) and (G – C)/(G + C) (Perna and Kocher 1995; Kan et al. 2016), respectively. The *MT-ND6* gene of *U. erythroryncha* mitogenome has strong skews of T versus A (–0.41), and a strong skew of C versus G (0.52). The other 12 protein-coding genes of the *U. erythroryncha* mitochondrial genome have a slight skew of A vs T (0 to 0.20), and a strong skew of C versus G (GC skew = –0.75 to –0.30).

The phylogenetic position of *U. erythroryncha* was estimated from a concatenated dataset based on 13 PCGs. Maximum likelihood (ML) analyses were performed with Raxml GUI v 1.3.1 (Silvestro and Michalak 2012; Zhang et al. 2012; Jiang et al. 2015). The complete mitochondrial DNA sequences have been used successfully to estimate phylogenetic relationships among Passeriformes. The results showed that *U. erythroryncha* and the other species of Corvidae were monophyletic group in this study. Furthermore, other genera of Corvidae (*Garrulus*, *Corvus*, *Pyrrhocorax*, *Nucifraga*, *Podoces*, *Pica*, *Cyanopica*) were basal to the balance of the Corvidae. Moreover, our results also support a sister-group relationship between Corvidae and Muscicapidae. They all appear 100% bootstrap value in ML analyses which is strongly supported. We speculate that there is still a complex phylogenetic relationship between finch and other families that is not known to us. This study will contribute to the phylogenetic analyses in Passeriformes.

**Figure 1.**
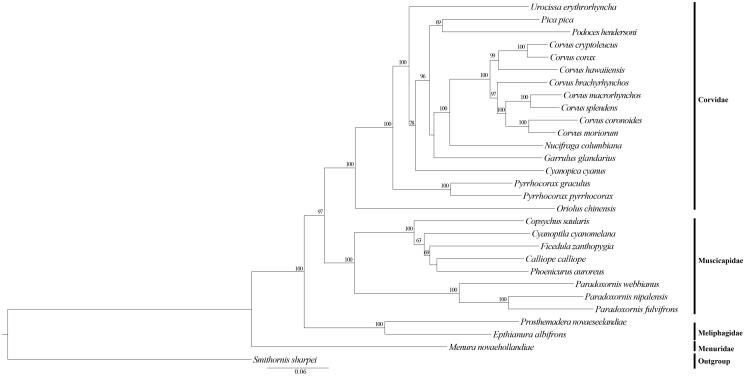
Phylogenetic tree of the relationships among Passeriformes based on the nucleotide dataset of the 13 PCGs. *Smithornis sharpei* was chosen as outgroup. ML analyses were implemented in ML + rapid bootstrap for 1000 replicates under GTRGAMMA, Branch lengths and topologies came from the ML analysis. All 29 species’ accession numbers are listed as below: *Corvus brachyrhynchos* NC_026461, *C. corax* NC_034838, *C. cryptoleucus* NC_034839, *C. coronoides* NC_035877, *C. hawaiiensis* NC_026783, *C. macrorhynchos* NC_027173, *C. moriorum* NC_031518, *C. splendens* NC_024607, *Cyanopica cyanus* NC_015824, *Garrulus glandarius* NC_015810, *Nucifraga columbiana* NC_022839, *Oriolus chinensis* NC_020424, *Pica pica* NC_015200, *Podoces hendersoni* NC_014879, *Pyrrhocorax graculus* NC_025927, *P. pyrrhocorax* NC_025926, *Urocissa erythroryncha* NC_020426, *Epthianura albifrons* NC_019664, *Prosthemadera novaeseelandiae* NC_029144, *Menura novaehollandiae* NC_007883, *Calliope calliope* NC_015074, *Copsychus saularis* NC_030603, *C. cyanomelana* NC_015232, *Ficedula zanthopygia* NC_015802, *Paradoxornis nipalensis* NC_028437, *P. webbianus* NC_024539, *Phoenicurus auroreus* NC_026066, *Smithornis sharpei* NC_000879.
